# Complex fibroblast response to glucocorticoids may underlie variability of clinical efficacy in the vocal folds

**DOI:** 10.1038/s41598-020-77445-9

**Published:** 2020-11-24

**Authors:** Ryosuke Nakamura, Shigeyuki Mukudai, Renjie Bing, Michael J. Garabedian, Ryan C. Branski

**Affiliations:** 1 Department of Rehabilitation Medicine, New York University Grossman School of Medicine, 240 East 38th Street, Suite 1774, New York, NY 10016 USA; 2 Department of Microbiology, New York University Grossman School of Medicine, New York, NY USA; 3 Department of Otolaryngology-Head and Neck Surgery, New York University Grossman School of Medicine, New York, NY USA

**Keywords:** Preclinical research, Molecular medicine

## Abstract

Similar to the hypertrophic scar and keloids, the efficacy of glucorticoids (GC) for vocal fold injury is highly variable. We previously reported dexamethasone enhanced the pro-fibrotic effects of transforming growth factor (TGF)-β as a potential mechanism for inconsistent clinical outcomes. In the current study, we sought to determine the mechanism(s) whereby GCs influence the fibrotic response and mechanisms underlying these effects with an emphasis on TGF-β and nuclear receptor subfamily 4 group A member 1 (NR4A1) signaling. Human VF fibroblasts (HVOX) were treated with three commonly-employed GCs+ /-TGF-β1. Phosphorylation of the glucocorticoid receptor (GR:NR3C1) and activation of NR4A1 was analyzed by western blotting. Genes involved in the fibrotic response, including *ACTA2, TGFBR1,* and *TGFBR2* were analyzed by qPCR. RNA-seq was performed to identify global changes in gene expression induced by dexamethasone. GCs enhanced phosphorylation of GR at Ser211 and TGF-β-induced *ACTA2* expression. Dexamethasone upregulated *TGFBR1*, and *TGFBR2* in the presence of TGF-β1 and increased active NR4A1. RNA-seq results confirmed numerous pathways, including TGF-β signaling, affected by dexamethasone. Synergistic pro-fibrotic effects of TGF-β were observed across GCs and appeared to be mediated, at least partially, via upregulation of TGF-β receptors. Dexamethasone exhibited diverse regulation of gene expression including NR4A1 upregulation consistent with the anti-fibrotic potential of GCs.

## Introduction

With the proliferation of office-based procedures for laryngeal disease, intralesional steroid injections for a variety of vocal fold pathology have concurrently increased. Although steroid use for inflammatory processes of the upper airway is ubiquitous, as described previously by our group, profound discrepancies emerge with regard to glucocorticoid (GC) use for vocal fold disease^1^. Furthermore, the outcomes of direct GC treatment for vocal fold fibrosis, qualitatively, appear disparate and similar to data in the keloid and hypertrophic scar literature. In keloids, for example, response rates following direct steroid injection vary from 50–100% with recurrence rates ranging from 9–50%^2–8^. More recent data suggested approximately 50% of keloids were GC resistant^9^. This variability in treatment response might be partially due to the divergent DNA binding capacity of glucocorticoid receptor (GR); whole-genome studies suggested GR‑binding sites on DNA vary substantially among tissues and cell types^10^. However, the underlying mechanism(s) is remains unclear.

Primarily, GCs are employed to reduce inflammation^10^ and numerous studies have reported the effects of GCs on leukocytes and vascular cells. Fibroblasts, the primary mediators of fibrotic tissue formation, also respond to GCs. Our laboratory recently reported an additive, pro-fibrotic effect of dexamethasone and transforming growth factor (TGF)-β on ACTA2 and SMAD7 mRNA expression in human vocal fold fibroblasts^11^. These data may provide insight into the variability in response to localized steroid injections for vocal fold fibrosis, acknowledging that GCs are an ideal pharmacological therapeutic in that they are inexpensive, FDA-approved, and amendable to injection into the larynx in the awake patient. However, GCs are diverse and one may hypothesize some degree of specificity with regard to the particular steroid chosen for a particular patient with a particular clinical presentation. Of note, a survey of otolaryngologists by our group suggested ‘*previous experience*’ and ‘*familiarity*’ were the primary factors underlying steroid selection for vocal fold pathology with less consideration for characteristics more likely to underlie therapeutic efficacy^1^.

Vocal fold fibrosis is characterized by altered extracellular matrix metabolism by fibroblasts within the lamina propria; activation of fibroblasts to the more metabolically active myofibroblasts is critical for initiation and maintenance of fibrosis. As such, therapies to address this aberrant metabolism should target the vocal fold fibroblast phenotype, initiated primarily via Transforming Growth Factor (TGF)-β. We, therefore, sought to elucidate potential interactions between TGF-β and GC signaling to provide insight into clinical variability with the ultimate goal of optimized therapeutic efficacy. Previously, our laboratory immunolocalized the GC receptor (GR) in the vocal fold mucosa in vivo^12^ and in our human vocal fold fibroblast cell line with an emphasis on three major serine phosphorylation sites within the N‐terminal region of the receptor involved in transcriptional regulation (Ser^203^, Ser^211^, and Ser^226^)^11^. In response to dexamethasone, Ser^211^ localized predominately to the nucleus^11,13,14^. And although this insight into more basic GC signaling provides a foundation for further investigation, the potential pro-fibrotic events associated with GCs warrant investigation to optimize clinical outcomes. As noted previously, we reported transcriptional changes suggestive of fibrosis in our human vocal fold fibroblast cell line when co-treated with TGF-β and dexamethasone concurrently^11^. In the current work, we sought to determine if these additive effects were consistent across the three most commonly employed GCs for laryngeal disease-dexamethasone, methylprednisolone, and triamcinolone. We also attempted to identify other potential mechanisms underlying these effects as well as the more favorable outcomes associated with GCs with an emphasis on TGF-β and nuclear receptor subfamily 4 group A member 1 (NR4A1) signaling to provide further foundational insight regarding optimal therapeutic strategies for patients with intractable vocal fold fibrosis.

## Results

### GCs altered GR phosphorylation

**Figure 1:**
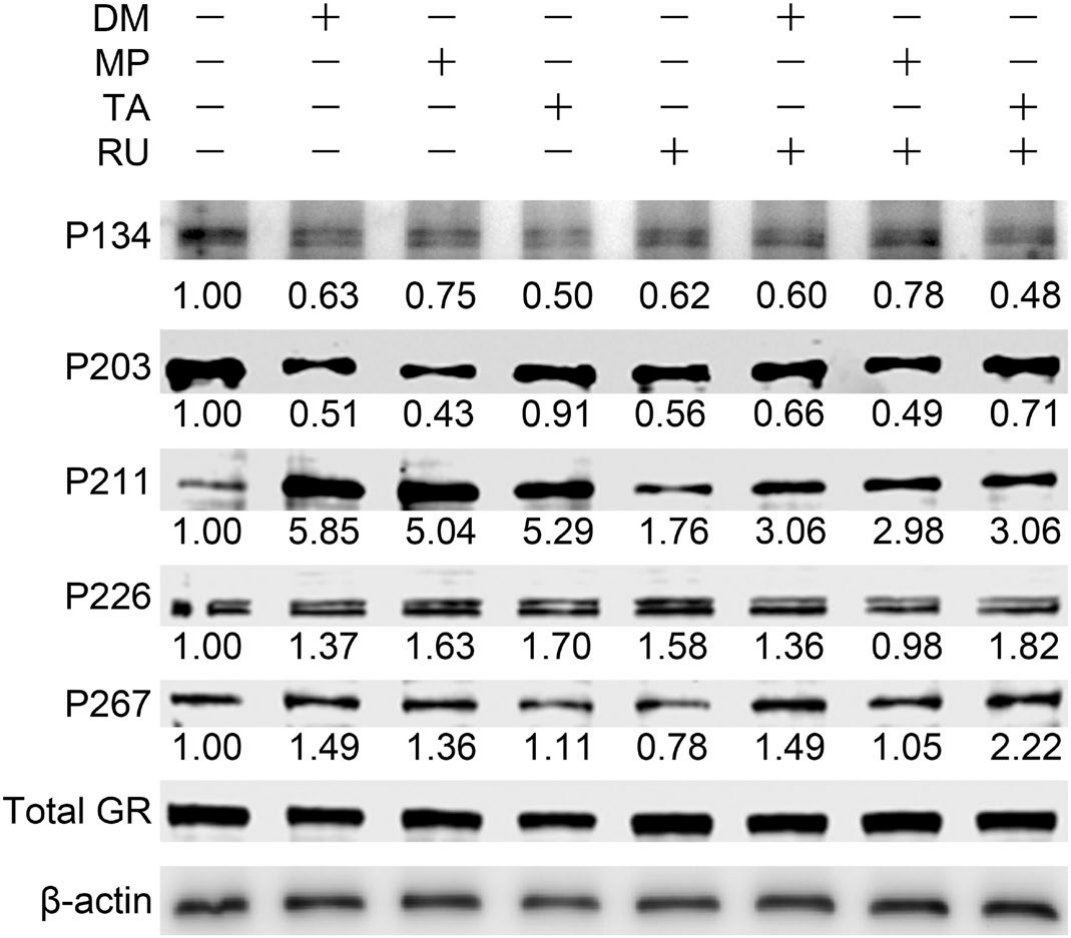
Phosphorylation of GR in vocal fold fibroblasts treated with glucocorticoids. HVOX cells were treated with dexamethasone (DM; 10^−7^ M), methylprednisolone (MP; 10^−5^ M), and triamcinolone (TA; 10^−5^ M) ± RU486 (RU; 10^−6^ M) for 1 h. GR phosphorylated at Ser134, 203, 211, 226, and 267 (P134, P203. P211. P226, P267) and total GR in cell lysates were examined by Western blotting. Intensities of the phosphorylated GR bands were normalized by the total GR band in each group, and relative intensities of the bands compared to the control group are shown below each band.

Human vocal fold fibroblasts (HVOX) were treated with dexamethasone, methylprednisolone, and triamcinolone ± RU486, a GR antagonist. All three GCs increased phosphorylation at Ser211, a site associated with GR transcriptional activity. Phosphorylation at Ser203, 226, and 267 were slightly affected (Fig. 1). All three GCs had a tendency to increase phosphorylation at Ser226 and 287 and to decrease phosphorylation at Ser134 and 203. GC-induced Ser211 phosphorylation was suppressed by RU486, consistent with previous reports^14^.

### GCs and TGF-β increased expression of pro-fibrotic genes including α-smooth muscle actin (ACTA2)

**Figure 2:**
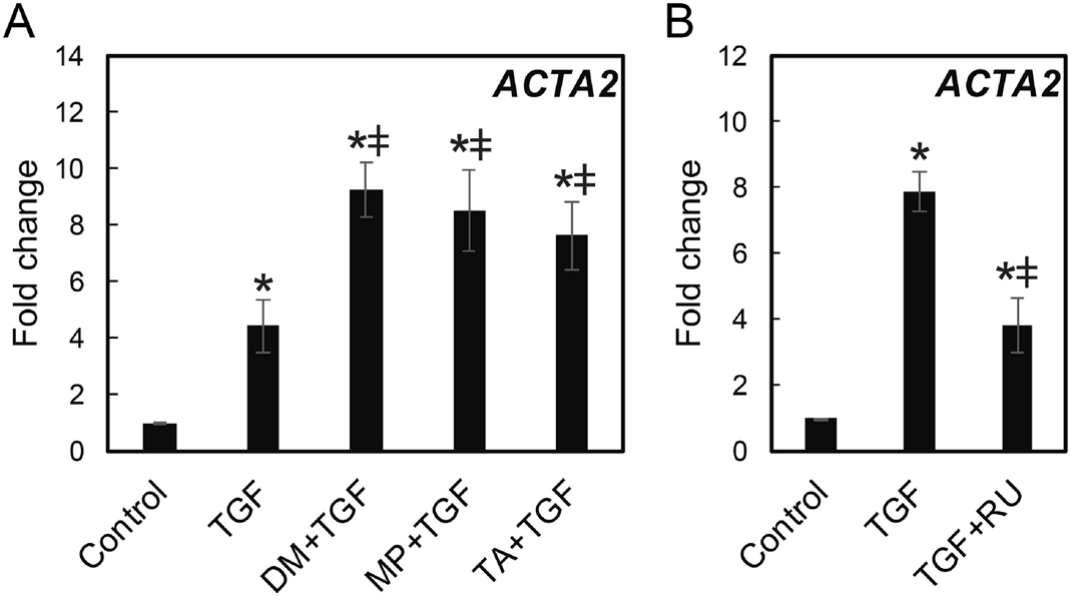
Expression levels of ACTA2 genes in TGF‐β1-induced vocal fold fibroblasts after glucocorticoids and RU486 administration. HVOX cells were treated with TGF-β1 (TGF-β1; 10 ng/mL), dexamethasone (DM; 10^−7^ M), methylprednisolone (MP; 10^−5^ M), triamcinolone (TA; 10^−5^ M), and/or RU486 (RU; 10^−6^ M) for 24 h (**A**,**B**), and ACTA2 mRNA expression was examined by qPCR. Relative expression levels of ACTA2 to GAPDH were compared with those of the control group, and are shown as mean ± SEM (n = 3). *P < 0.05 vs. control. ^ǂ^*P* < 0.05 vs TGF-β1.

**Figure 3:**
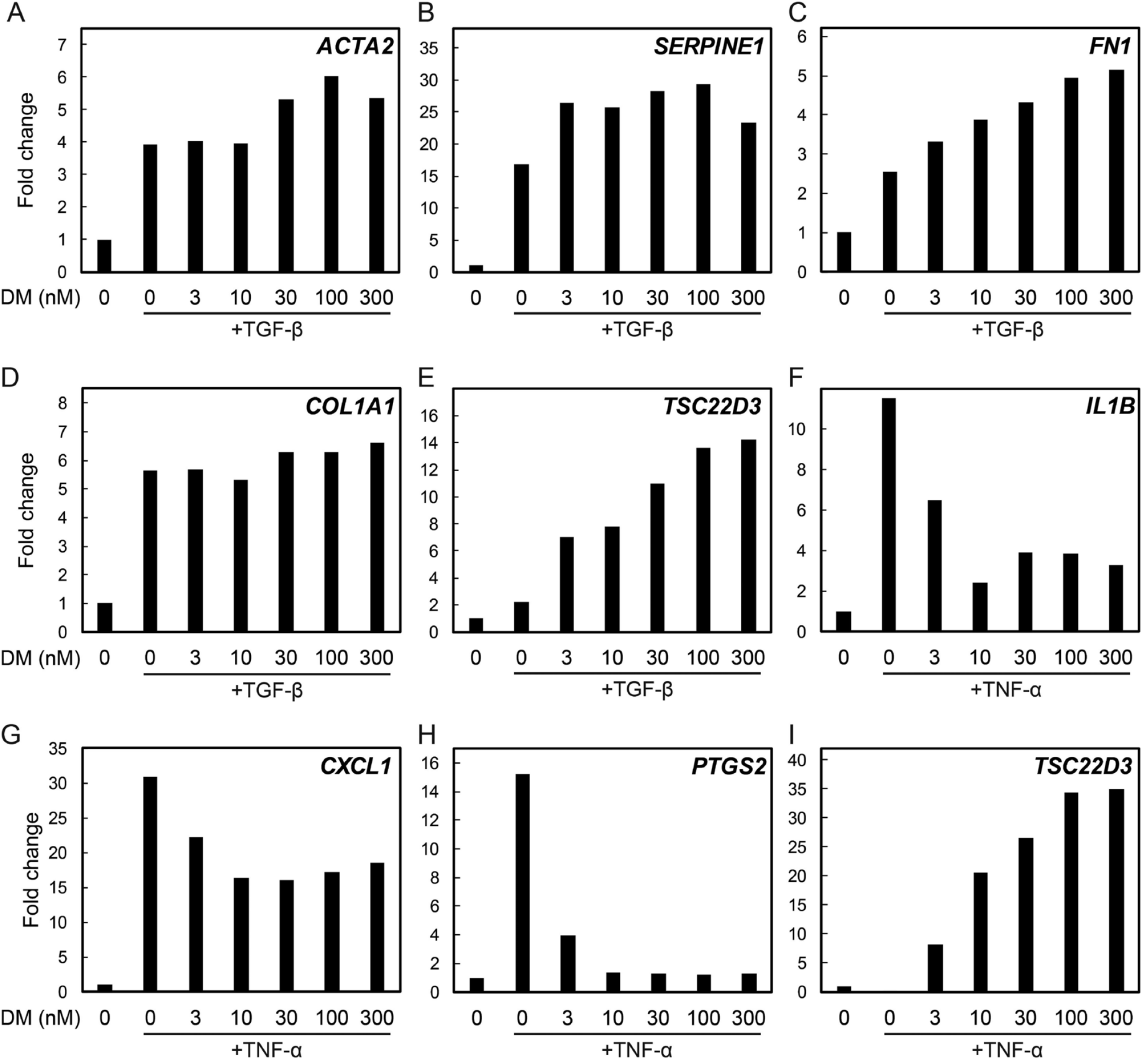
Fibrotic, inflammatory, and *TSC22D3* genes expression in human vocal fold fibroblasts after treatment with TGF-β1, TNF-α, and dexamethasone. HVOX cells were treated with TGF-β1 (TGF-β; 10 ng/mL), TNF-α (10 ng/mL), and dexamethasone (DM; 0-100 nM) alone or in combination for 24 h (**A**,**B**), and *ACTA2* (**A**), *SERPINE1* (**B**), *FN1* (**C**), *COL1A1* (**D**), *TSC22D3* (**E**,**I**), *IL1B* (**F**), *CXCL1* (**G**), *PTGS2* (**H**) mRNA expression was examined by qPCR. Relative expression levels to *GAPDH* were compared with those of an untreated group.

HVOX were treated with GCs, RU486, and/or TGF-β1 and *ACTA2* mRNA expression was analyzed by qPCR. TGF-β1 treatment upregulated *ACTA2* expression and concomitant treatment with GCs and TGF-β1 further increased expression (Fig. 2A). Inhibition of GR via RU486 reduced TGF-β-induced *ACTA2* expression (Fig. 2B). *ACTA2* expression was concentration-dependent (Fig. 3A). *SERPINE1* and *FN1*, both pro-fibrotic mediators^15,16^, were also upregulated in response to dexamethasone in a concentration-dependent manner (Fig. 3B,C). *COL1A1* mRNA was slightly upregulated by dexamethasone (Fig. 3D). Expression of these genes tended to peak at ~ 100 nM dexamethasone, similar to expression of *TSC22D3,* which is regulated by GR binding to GR-responsive element in the promoter region (Fig. 3E). To examine the effects of GCs on inflammatory genes in HVOX, tumor necrosis factor-α (TNF-α), a prototypic pro-inflammatory cytokine, was employed + /- dexamethasone^17,18^. As anticipated, expression of inflammatory genes, *IL-1β*, *CXCL1*, and *PTGS2* was upregulated by TNF-α; this response was suppressed by DM in a concentration-dependent manner (Fig. 3F–H). Optimal suppression was observed at 10 nM DM, markedly less than the most effective concentration to stimulate pro-fibrotic gene expression (Fig. 3E,I).

### Dexamethasone altered TGF-β signaling and receptor expression

**Figure 4:**
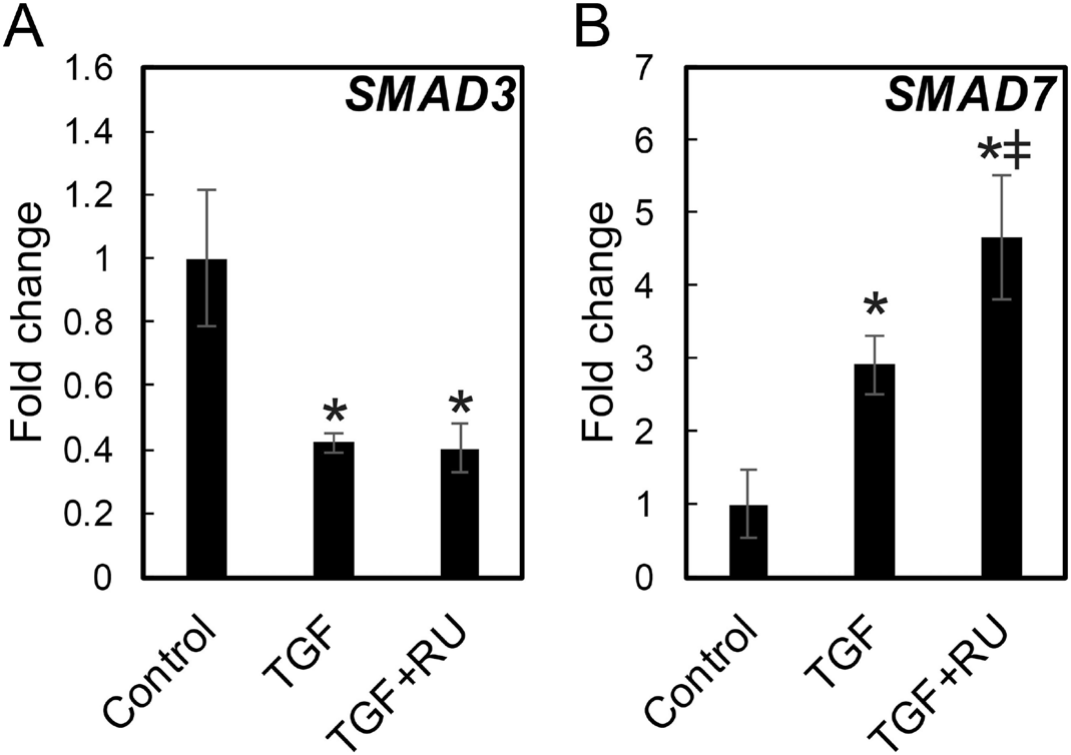
Expression levels of *SMAD3* and *SMAD7* genes in vocal fold fibroblasts after TGF‐β1 and RU486 administration. HVOX cells were treated with transforming growth factor-β1 (TGF-β; 10 ng/mL), dexamethasone (DM; 10^−7^ M), and/or RU486 (RU; 10^−6^ M) for 24 h. mRNA levels for smad3 (**A**) and smad7 (**B**) were examined by quantitative real-time polymerase chain reaction. Relative expression levels of each gene to *GAPDH* were compared with those of the control group, and are shown as mean ± SEM (n = 3). *P < 0.05 vs. control. ^ǂ^*P* < 0.05 vs. TGF-β.

**Figure 5:**
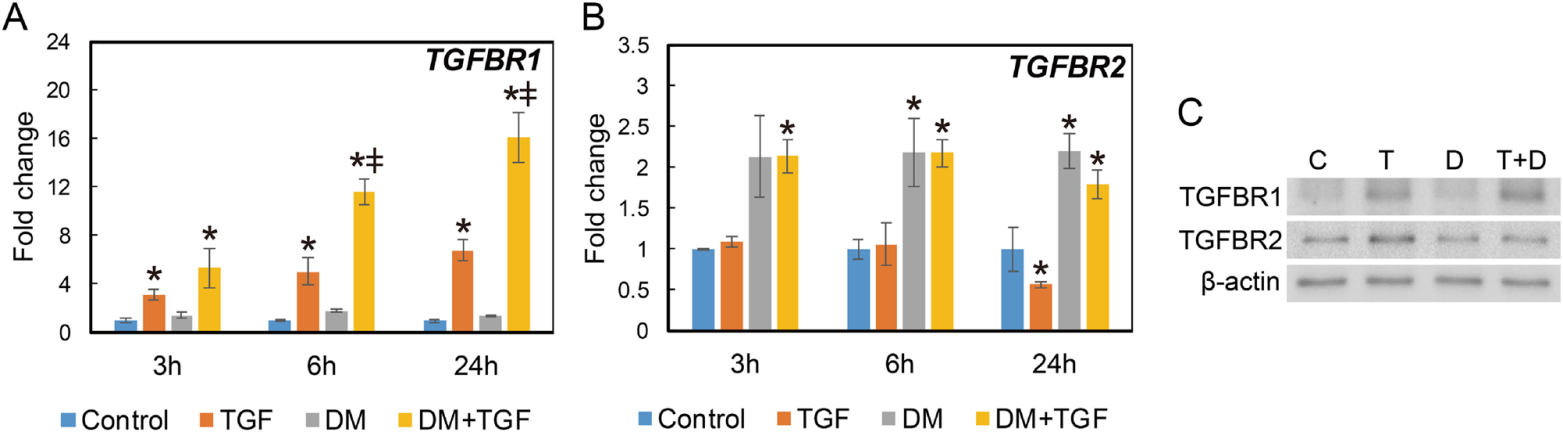
Expression levels of TGF-β receptors in vocal fold fibroblasts after treatment with TGF-β and dexamethasone. HVOX cells were treated with TGF-β1 (TGF-β; 10 ng/mL), dexamethasone (DM; 10^−7^ M), and RU486 (RU; 10^−6^ M) for 3, 6, and 24 h. *TGFBR1* and *TGFBR2* mRNA expression was examined by qPCR (**A**,**B**). Relative expression levels of each gene to *GAPDH* were compared with those of the control group, and are shown as mean ± SEM (n = 3). *P < 0.05 vs. control. ^ǂ^*P* < 0.05 vs. TGF-β. TGFBR1 and TGFBR2 protein expression in the cells treated for 24 h were examined by Western blotting (**C**).

*SMAD3* and *SMAD7* mRNA expression, in response to TGF-β1 ± RU486, was analyzed by qPCR. TGF-β downregulated *SMAD3* expression and upregulated SMAD7 expression (Fig. 4A,B). RU486 had no effect on *SMAD3* expression. However, RU486 increased *SMAD7* expression (Fig. 4A,B). TGF-β receptor expression was also investigated in response to dexamethasone. *TGFBR1* mRNA expression increased slightly in response to 6 h of dexamethasone exposure (Fig. 5A). However, in the presence of TGF-β1, dexamethasone significantly increased *TGFBR1* mRNA expression. *TGFBR2* mRNA expression was also upregulated by dexamethasone, independent of TGF-β1 supplementation (Fig. 5B). Western blotting confirmed co-treatment with TGF-β1 and dexamethasone increased TGFBR1 protein levels compared to TGF-β1 alone (Fig. 5C). TGFBR2 was not altered by dexamethasone.

### Dexamethasone altered NR4A1 expression and phosphorylation

**Figure 6:**
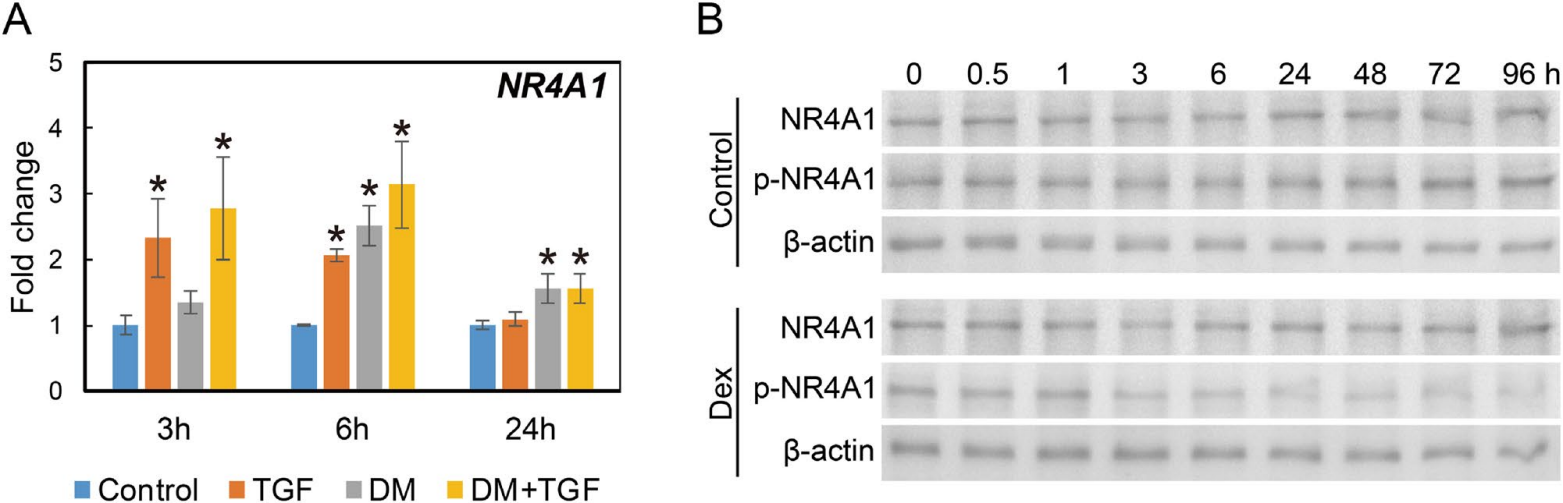
NR4A1 expression and phosphorylation in Dexamethasone-treated vocal fold fibroblasts. HVOX cells were treated with TGF-β1 (TGF-β; 10 ng/mL) and dexamethasone (DM; 10^−7^ M) for 3, 6, and 24 h. *NR4A1* mRNA expression was examined by qPCR (**A**). Relative expression levels of each gene to *GAPDH* were compared with those of the control group, and are shown as mean ± SEM (n = 3). *P < 0.05 vs. control. ^ǂ^*P* < 0.05 vs. TGF-β. HVOX cells were treated with dexamethasone for 0, 0.5, 1, 3, 6, 24, 48, 72 and 96 h and Western blot was performed using antibodies against NR4A1 and phosphorylated NR4A1 (p-NR4A1) (**B**).

Dexamethasone increased *NR4A1* expression (Fig. 6A). However, this response was temporally delayed compared to TGF-β. Dexamethasone-induced *NR4A1* upregulation was sustained for 24 h prior to attenuation. NR4A1 protein levels appeared similar in both dexamethasone-treated and control cells (Fig. 6B). However, phosphorylation of NR4A1 at Ser351 was reduced in dexamethasone treated cells.

### Transcriptome analysis of dexamethasone treated HVOX cells

**Figure 7:**
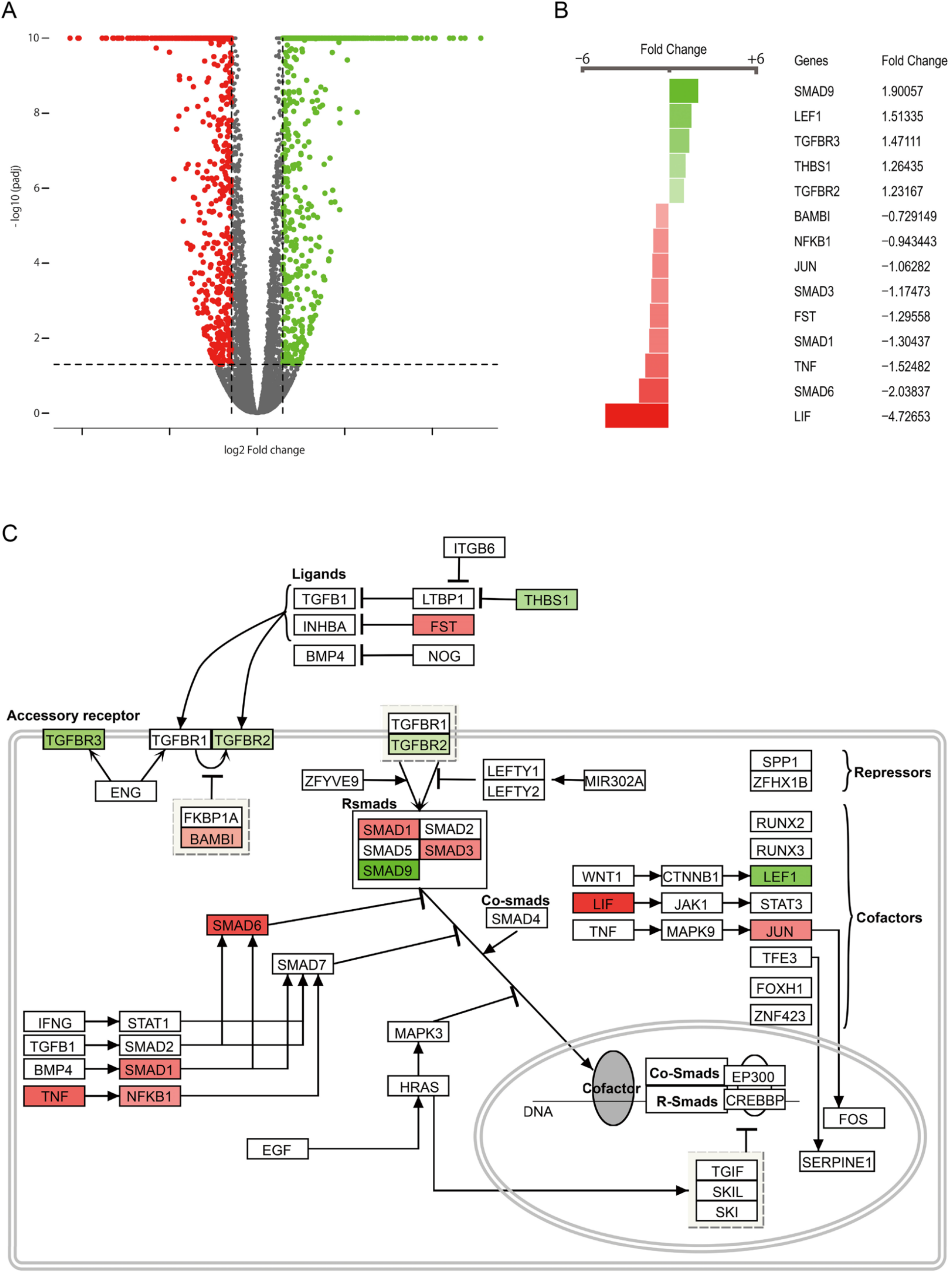
Alteration of the transcriptome induced by dexamethasone-treatment in vocal fold fibroblasts. HVOX cells were treated with dexamethasone (DM; 10^−7^ M) for 4 h. RNA-seq was performed. Data were analyzed using ROSALIND. Changes in expression levels at ≥ t1.5 and ≤ − 1.5 fold with p-value at < 0.05 were determined significant. Changes in expression level and p-value after dexamethasone treatment are depicted as a volcano plot (**A**). A heat map of genes related to TGF-β signaling as described in WikiPathways (**B**). A schematic of TGF-β receptor signaling in WikiPathways (https://www.wikipathways.org/index.php/Pathway:WP560) (**C**). Genes significantly upregulated or downregulated by dexamethasone are shown as green and red, respectively, in the volcano plot, heat map, and schematic.

RNA-seq in HVOX cells treated with vehicle or dexamethasone was performed to provide a more global sense for the effects of GCs. Approximately 1,400 genes were modulated by dexamethasone (p-value: < 0.05; Fold change: ≤ − 1.5, ≥ 1.5; Fig. 7A). Dexamethasone altered expression of genes associated with various signaling pathways registered in WikiPathways. Table 1 lists 20 pathways with the lowest p-values, according to WikiPathways. This list includes pathways related to adipogenesis, inflammation, fibrosis, extracellular matrix metabolism, and TGF-β receptor signaling. Figure 7B,C shows genes related to TGF-β receptor signaling altered by dexamethasone. *TGFBR2* upregulation and *SMAD3* downregulation were detected, consistent with our findings. Pathway analysis was then performed, focusing only on up- or downregulated genes (Tables 2 and 3). Upregulated genes were primarily associated with inflammatory and immune responses. Similar trends were observed with downregulated genes.

**Table 1:** Twenty pathways with the lowest p-values, according to WikiPathways, including pathways related to adipogenesis, inflammation, fibrosis, extracellular matrix metabolism, and TGF-β receptor signaling.

Term	p value	Genes in term	Target genes in term	Num up	Num down
Transcription factor regulation in adipogenesis	3.59E−06	22	11	6	5
Photodynamic therapy-induced NF-kB survival signalling	4.63E−06	35	14	0	14
Apoptosis-related network due to altered Notch3 in ovarian cancer	1.97E−05	54	17	4	13
Adipogenesis	2.28E−05	131	30	16	14
Lung fibrosis	4.89E−05	63	18	7	11
TNF related weak inducer of apoptosis (TWEAK) signaling pathway	5.26E−05	42	14	2	12
Prostaglandin synthesis and regulation	1.27E−04	30	11	5	6
Apoptosis	1.48E−04	86	21	5	16
Overview of nanoparticle effects	3.62E−04	19	8	2	6
Matrix metalloproteinases	6.26E−04	30	10	4	6
Mesodermal commitment pathway	6.34E−04	156	30	14	16
Cytokines and inflammatory response	8.58E−04	26	9	1	8
Spinal cord injury	1.09E−03	119	24	8	16
Development and heterogeneity of the ILC family	1.11E−03	32	10	5	5
Senescence and autophagy in cancer	1.17E−03	106	22	6	16
Nuclear receptors	1.62E−03	39	11	6	5
Hepatitis C and hepatocellular carcinoma	1.75E−03	51	13	2	11
Regulation of toll-like receptor signaling pathway	1.92E−03	145	27	3	24
TGF-beta receptor signaling	2.08E−03	58	14	5	9
EBV LMP1 signaling	2.20E−03	24	8	1	7

**Table 2:** Pathway analysis of upregulated genes.

Term	p value	Genes in term	Target genes in term	Num up
Adipogenesis	3.31E−04	131	16	16
Transcription factor regulation in adipogenesis	3.78E−04	22	6	6
Nuclear receptors	8.51E−03	39	6	6
Endochondral ossification	9.88E−03	65	8	8
Small ligand GPCRs	1.02E−02	19	4	4
Prostaglandin synthesis and regulation	1.14E−02	30	5	5
Copper homeostasis	1.18E−02	54	7	7
NAD metabolism, sirtuins and aging	1.24E−02	11	3	3
Mesodermal commitment pathway	1.32E−02	156	14	14
Development and heterogeneity of the ILC family	1.50E−02	32	5	5
BMP2-WNT4-FOXO1 pathway in human primary endometrial stromal cell differentiation	2.01E−02	13	3	3
Retinoblastoma (RB) in cancer	2.03E−02	88	9	9
Alpha 6 beta 4 signaling pathway	2.16E−02	35	5	5
Wnt/beta-catenin signaling pathway in leukemia	2.32E−02	24	4	4
Lung fibrosis	2.59E−02	63	7	7
Hypothesized pathways in pathogenesis of cardiovascular disease	2.67E−02	25	4	4
Zinc homeostasis	2.69E−02	37	5	5
Cardiac progenitor differentiation	3.50E−02	53	6	6
Angiopoietin like protein 8 regulatory pathway	4.23E−02	132	11	11
Focal adhesion	4.65E−02	1	1	1

**Table 3:** Pathway analysis of downregulated genes.

Term	p value	Genes in term	Target genes in term	Num down
Photodynamic therapy-induced NF-kB survival signalling	2.92E−09	35	14	14
Regulation of toll-like receptor signaling pathway	2.40E−06	145	24	24
TNF related weak inducer of apoptosis (TWEAK) signaling pathway	2.78E−06	42	12	12
RIG-I-like receptor signaling	5.77E−06	60	14	14
Apoptosis-related network due to altered Notch3 in ovarian cancer	8.66E−06	54	13	13
Toll-like receptor signaling pathway	2.12E−05	103	18	18
Apoptosis	2.74E−05	86	16	16
Type II interferon signaling (IFNG)	3.28E−05	37	10	10
Cytokines and inflammatory response	7.46E−05	26	8	8
TNF alpha signaling pathway	8.50E−05	94	16	16
Photodynamic therapy-induced AP-1 survival signalling	1.27E−04	51	11	11
Hepatitis C and hepatocellular carcinoma	1.27E−04	51	11	11
Apoptosis modulation and signaling	2.97E−04	94	15	15
EBV LMP1 signaling	3.09E−04	24	7	7
Senescence and autophagy in cancer	3.61E−04	106	16	16
Overview of nanoparticle effects	5.26E−04	19	6	6
Lung fibrosis	8.76E−04	63	11	11
Spinal cord injury	1.32E−03	119	16	16
TLR4 signaling and tolerance	1.34E−03	30	7	7
Differentiation pathway	1.52E−03	48	9	9

## Discussion

Vocal fold scarring poses a significant clinical challenge. Scarring and the associated tissue stiffness result in decreased vibratory pliability of the vocal fold mucosa and often underlie aberrant voice quality and resultant voice-related disability with profound socioeconomic implications^19–21^. Direct GC injection to regions of vocal fold fibrosis has become an increasingly common therapeutic option with emerging, yet variable data regarding efficacy^22–24^. This response variability provides an ideal platform for mechanistic investigation. Although GCs hold profound utility for inflammatory conditions of the upper airway, the value of GCs for fibroplastic processes is less clear, particularly given the unique biophysical demands placed upon the vocal folds. The current investigation sought to address this issue with a keen eye towards the value of a more *personalized* approach to GC use for vocal fold pathology; variable efficacy may be associate with patient- and/or glucocorticoid- related factors. Mechanistically, we postulate that the pro-fibrotic effects of GCs are enhanced by endogenous TGF-β, putatively exclusive to specific patients. To support this hypothesis, recent data suggest GC-responders and non-responders in patients with keloids have different histological and gene expression features, particularly with regard to glucocorticoid receptor profiles^25^.

Our laboratory recently immunolocalized GR in the vocal fold mucosa and our immortalized human vocal fold fibroblast cell line (HVOX)^11^. In the currently study, three commonly employed GCs stimulated GR phosphorylation at Ser211 in HVOX cells. This site influences both activation and repression of GR target genes^26^. Downstream, TGF-β-induced *ACTA2* expression was enhanced by all three GCs and suppressed by RU486. Upstream, combined treatment with TGF-β1 and dexamethasone yielded increased TGF-β receptor1 (TGFBR1) gene expression compared to TGF-β1 alone. Similar findings were observed for TGFBR1 translation. Although less robust, TGF-β receptor2 (TGFBR2) expression also increased in response to dexamethasone. These data suggest GCs enhance TGF-β signaling, similar to a recent report in prostate cancer cells^27^. In vivo, subcutaneous injection of dexamethasone increased TGFBR1 and decreased TGFBR2 in wounded skin. In contrast, however, dexamethasone had no effect on TGF-β receptor expression in hepatic stellate cells^28,29^. Of note, the response to all three GCs was relatively consistent.

These data suggest TGF-β and GC signaling synergistically stimulate the myofibroblastic phenotype. Interestingly, these data imply that *inhibition* of GR may be anti-fibrotic, and provide insight into the variability in clinical response to GCs in laryngology. *ACTA2* downregulation by RU486 might partially rely on the negative feedback of TGF-β/SMAD signaling pathway; RU486 upregulated *SMAD7* expression. These findings conflict with recent data suggesting GCs reduced ACTA2 expression in human normal skin and keloid fibroblasts^30^. However, this disparate findings concur with our hypotheses regarding tissue and/or patient specificity with regard to the anti-fibrotic actions of GCs^10,31^.

In this context, we hypothesize that GCs may limit fibrosis by stimulating NR4A1. NR4A1 is an orphan nuclear receptor and is involved in multiple cellular events^32,33^. Recent evidence confirmed an inhibitory role of NR4A1 in TGF-β signaling and tissue fibrosis^34^. Our group also reported increased NR4A1 expression following vocal fold injury in a rodent model and NR4A1 inhibited the induction of a pro-fibrotic phenotype by TGF-β1 in human vocal fold fibroblasts^35^. In the current study, dexamethasone increased NR4A1 expression and interestingly, decreased phosphorylation of NR4A1 at Ser351. Ser351-phosphorylation has been shown to disrupt transcriptional activity of NR4A1 and attenuate inhibition of TGF-β signaling^34,36^. These data suggest provide some mechanistic insight regarding the anti-fibrotic outcomes associated with GCs, even in the context of increased *ACTA2* expression.

GCs are primarily used to reduce inflammation via broad effects across cell types including via a phenotypic shift of macrophages, which may affect the fibrotic responses. M2 macrophages, an alternatively activated phenotype, stimulate fibroblasts^37^ and GCs promote macrophage polarization to the M2c subset with increased expression of scavenger proteins (CD163 and CD206), anti-inflammatory cytokine IL-10, and TGF-β^10^. However, implantation of M2c macrophages improved lung and kidney fibrosis in mouse models^38,39^. The effects of GCs on non-fibroblast cells are complex and investigation of infiltrating leukocytes in the VFs is certainly warranted. As anticipated, dexamethasone had an inhibitory effect on pro-inflammatory gene expression. Of note, however, the concentration of dexamethasone effective on fibrotic gene upregulation and inflammatory gene suppression varied. These data imply the potential for an optimal therapeutic window for GCs might be critical to address vocal fold inflammation. Mechanisms underlying this variable response are unclear from the current data. However, GR affinity to transcription factors is likely. GR is known to modulate other transcription factors via multiple mechanism, including direct binding to GR responsive elements, tethering of other proteins, and binding to GR responsive elements as protein complexes. The bioavailability of transcriptional factors involved in inflammation may be increased related to those associated with fibrotic gene expression^10^.

Consistent with our hypothesis that the effects of GCs are immensely diverse, RNASeq analysis found dexamethasone altered the expression of ~ 1400 genes. Dexamethasone regulated pathways related to adipogenesis, inflammation, fibrosis, ECM metabolism, and several developmental events. These data suggest GC signaling is involved not only in inflammation and glucose metabolism, but many other cellular events. Consistent with our data, TGFBR2 upregulation was observed via RNA-seq and TGF-β receptor signaling was in the top 20 pathways affected by dexamethasone. These data further confirm that TGF-β signaling is a major target of GCs.

In summary, three commonly employed GCs enhanced TGF-β-induced *ACTA2* expression in human vocal fold fibroblasts and this effect appeared to be mediated, at least partially, via upregulation of TGF-β receptors. However, this response is likely complex as dexamethasone expression of many genes in human vocal fold fibroblasts and decreased phosphorylation of NR4A1, suggesting profound diversity with regard to the actions of GCs. This diversity putatively underlies the variable clinical outcomes and likely provides an opportunity to optimize outcomes based on a more personalized approach to GC therapy. The sheer diversity of the genetic response to GCs is profound and clinical efficacy is likely to be related to many factors. Ideally, further identification of the relevant factors determining efficacy is required in addition to robust screening techniques to quantify these biological phenomena in order to optimize treatment approaches. The current study is an initial step in this regard.

## Methods

### Cell culture

An immortalized human vocal fold fibroblast cell line created in our laboratory was employed for all experimentation. This cell line, referred to as HVOX, has been shown to be stable through multiple population doublings. Cells in passages 11–20 were used. Cells were maintained in Dulbecco's Modified Eagle's Medium (DMEM) containing 10% fetal bovine serum and 1% antibiotic/antimycotic (Life Technologies, Grand Island, NY) at 37 °C under standard cell culture conditions. Following overnight serum starvation using FBS-free DMEM, cells were treated with dexamethasone, methylprednisolone, triamcinolone, RU486 (Sigma-Aldrich, Massachusetts, MA), TGF-β1 (10 ng/mL; Life Technologies), and TNF-α alone or in combination.

### Western blotting

Following treatment, total cellular protein was extracted using Mammalian Protein Extraction Reagent (Thermo Scientific, Waltham, MA) supplemented with Halt Protease Inhibitor Cocktail (Thermo Scientific), 5 mM EDTA Solution (Thermo Scientific), Calyculin A (Cell Signaling), and 2-mercaptoethanol (Life Technologies). Each protein lysate was loaded on 8% sodium dodecyl sulfate–polyacrylamide gels and then transferred to PVDF membranes (Invitrogen) and blocked with 5% BSA (Fisher Scientific) overnight at 4 °C. Membranes were incubated with primary antibodies against GR phosphorylation sites (using human GR number scheme) S134, S203, S211, S226, and S267 (1:1000)^14,15^; total GR (1:1000; #3660, Cell Signaling); TGF-β receptor 1 (TGRBR1,1:1000; #PA5-32631, Thermo Scientific, Waltham, MA); TGF-β receptor 2 (TGFBR2, 1:1000; #ab186838, Abcam, Cambridge, UK); phosphorylated NR4A1 (1:1000; #5095, Cell Signaling), and total NR4A1 (1:1000; #3960, Cell Signaling) for 48 h or β-actin (1:5000; #4970, Cell Signaling) for 1 h at 4 °C followed by 1-h incubation with IRDye (LI-COR) secondary antibody (1:10,000; #925-32211, LI-COR, Lincoln, NE) or horseradish peroxidase-conjugated secondary antibody (1:20,000; #7074, Cell Signaling). IRDye was detected with the Odyssey CLx Imaging system (LI-COR)*.* Horseradish peroxidase was detected using ChemiDoc MP (Bio-Rad Laboratories, Hercules, CA) after incubation with SuperSignal™ West Dura Extended Duration Substrate (Pierce Biotechnology, Rockford, IL).

### Quantitative real-time polymerase chain reaction (qPCR)

Cells were harvested at 3, 6, and 24 h after the treatment. Total RNA was extracted via the RNeasy Mini Kit (Qiagen, Valencia, CA) and reverse transcribed with a High-Capacity cDNA Reverse Transcription Kit (Applied Biosystems). The TaqMan Gene Expression kit (Life Technologies) and StepOne Plus (Applied Biosystems) were employed for quantitative analyses. Taqman primer probes for *SMAD3* (Hs00969210_m1), *SMAD7* (Hs00998193_m1), *ACTA2* (Hs00426835_g1), *NR4A1* (Hs00374226_m1), *COL1A1* (Hs00164004_m1), *FN1* (Hs01549976_m1), *SERPINE1* (Hs00167155_m1), *TSC22G3* (Hs00608272_m1), *IL1B* (Hs01555410_m1), *CXCL1* (Hs00236937_m1), *PTGS2* (Hs00153133_m1), and *GAPDH* (Hs02758991_g1) were employed. The ΔΔCt method was employed with GAPDH as the housekeeping gene for quantification of relative expression.

### RNA-sequencing (RNA-seq)

Cells were harvested 4 h after treatment. Total RNA was isolated by using the RNeasy Mini Kit and RNA-seq libraries were prepared using the Illumina TruSeq stranded mRNA kit with 10 cycles of PCR amplification, starting from 500 ng of total RNA (DNAse I-digested). Amplified libraries were purified using AMPure beads, quantified by Qubit and QPCR, and visualized in an Agilent Bioanalyzer. The indexed libraries were pooled equimolarly and run on an Illumina HiSeq 4000 as single, 50 nucleotide in length (3 lanes total). Raw reads and data analysis were performed on Rosalind (OnRamp Bioinformatics Genomics Research Platform, OnRamp Bioinformatics, San Diego, CA). Quality scores were evaluated using FastQC tool. Human genome build hg19 was used as the reference. HTseq was used for quantification of individual sample reads. DEseq2 was applied to normalize the reads via relative long expression and to determine fold changes and p-values. Functional enrichment analysis of pathways referenced from WikiPathways via HOMER. Figure 7C was designed using Wikipathways, an open access site. This figure was derived from the TGF-β Receptor Signaling (Homo Sapiens; https://www.wikipathways.org/index.php/Pathway:WP560) (Supplementary information S1).

### Statistical considerations

Western blotting and qPCR analyses were performed in triplicate, at least. For statistical comparisons, Tukey's honestly significant difference tests were employed and *p* ≤ 0.05 was considered significant.

## Supplementary information

Supplementary Information 1.

Supplementary Information 2.

Supplementary Information 3.

## Data Availability

All data study can be obtained from the corresponding author upon reasonable request.
