# Supplementary figures and images for: Complex fibroblast response to glucocorticoids may underlie variability of clinical efficacy in the vocal folds
Source: Sci Rep. 2020 Nov 24;10:20458. doi: 10.1038/s41598-020-77445-9 (PMC7686477; doi:10.1038/s41598-020-77445-9)

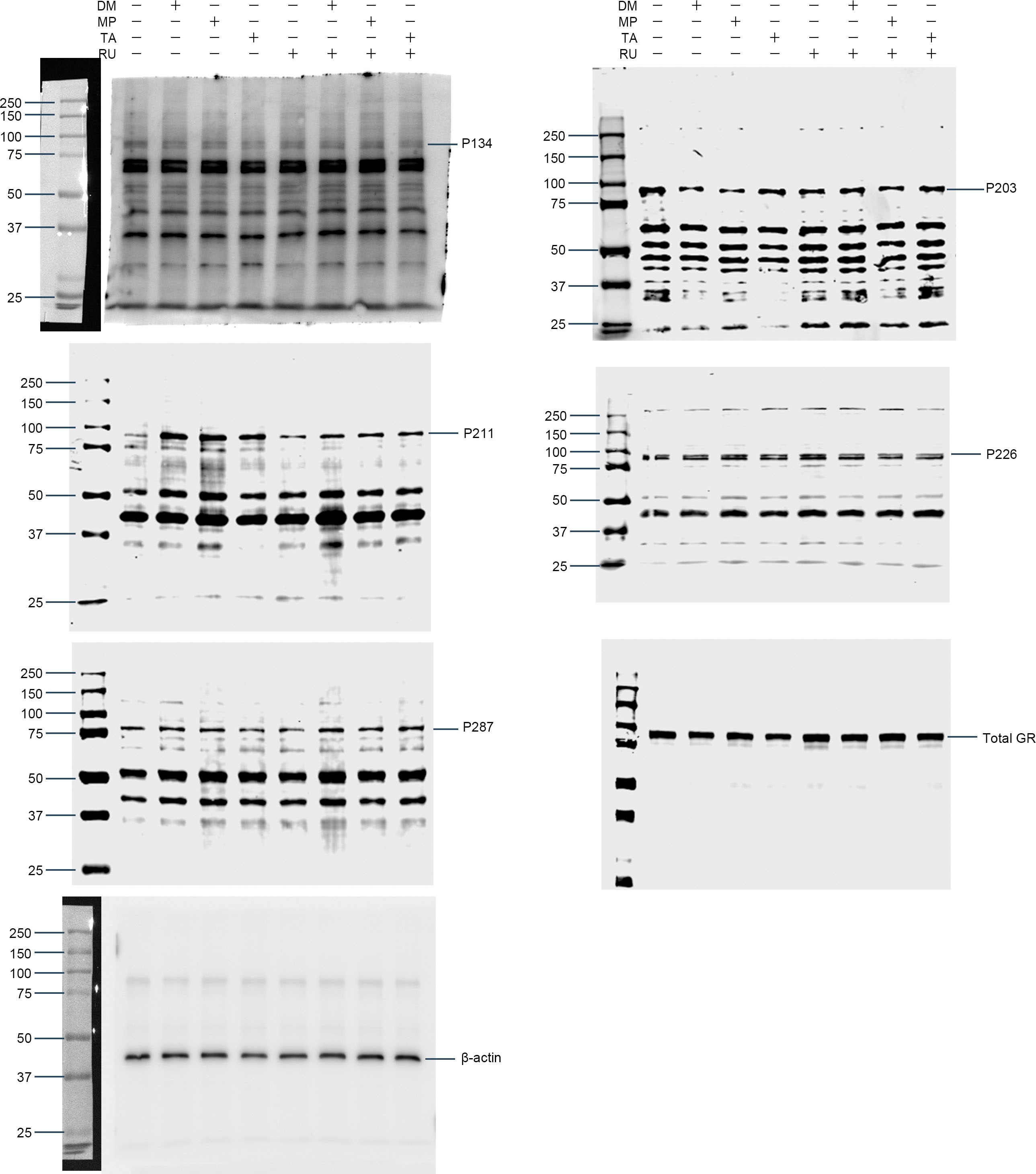

Supplement: Supplementary file 1 — Supplementary Information 1. [file 41598_2020_77445_MOESM1_ESM.jpg]

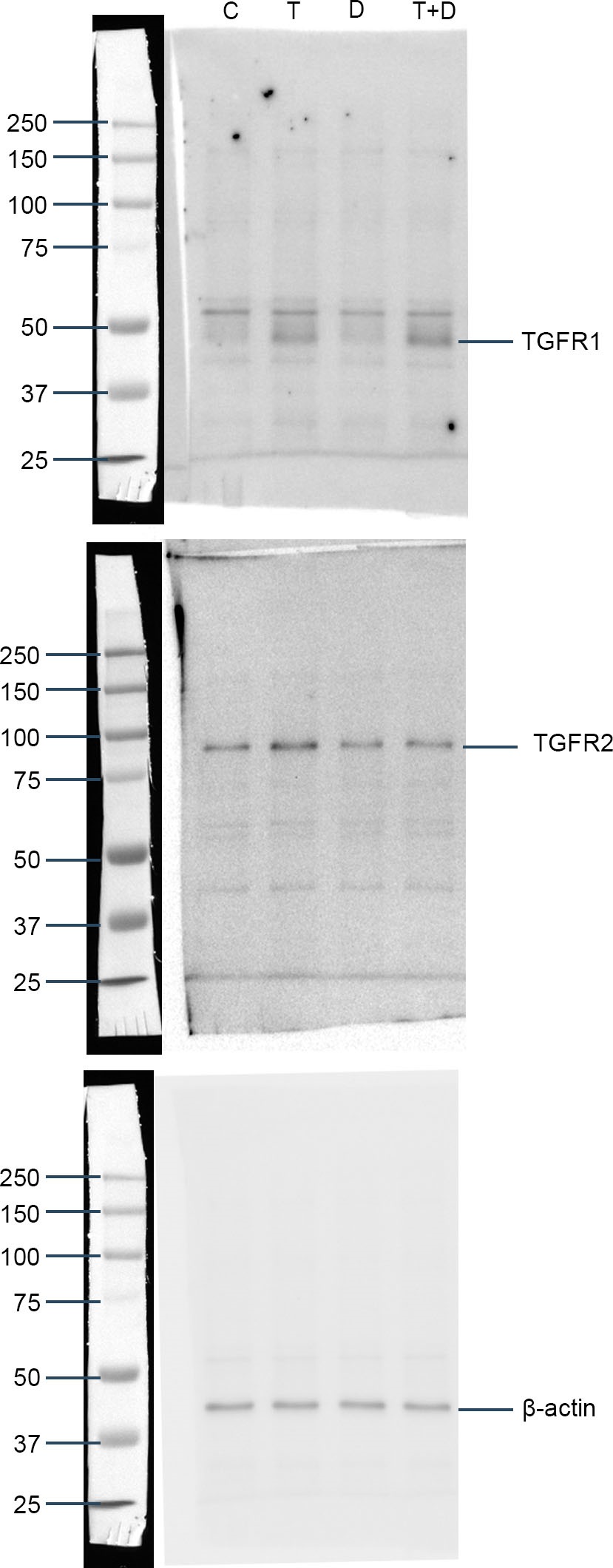

Supplement: Supplementary file 2 — Supplementary Information 2. [file 41598_2020_77445_MOESM2_ESM.jpg]

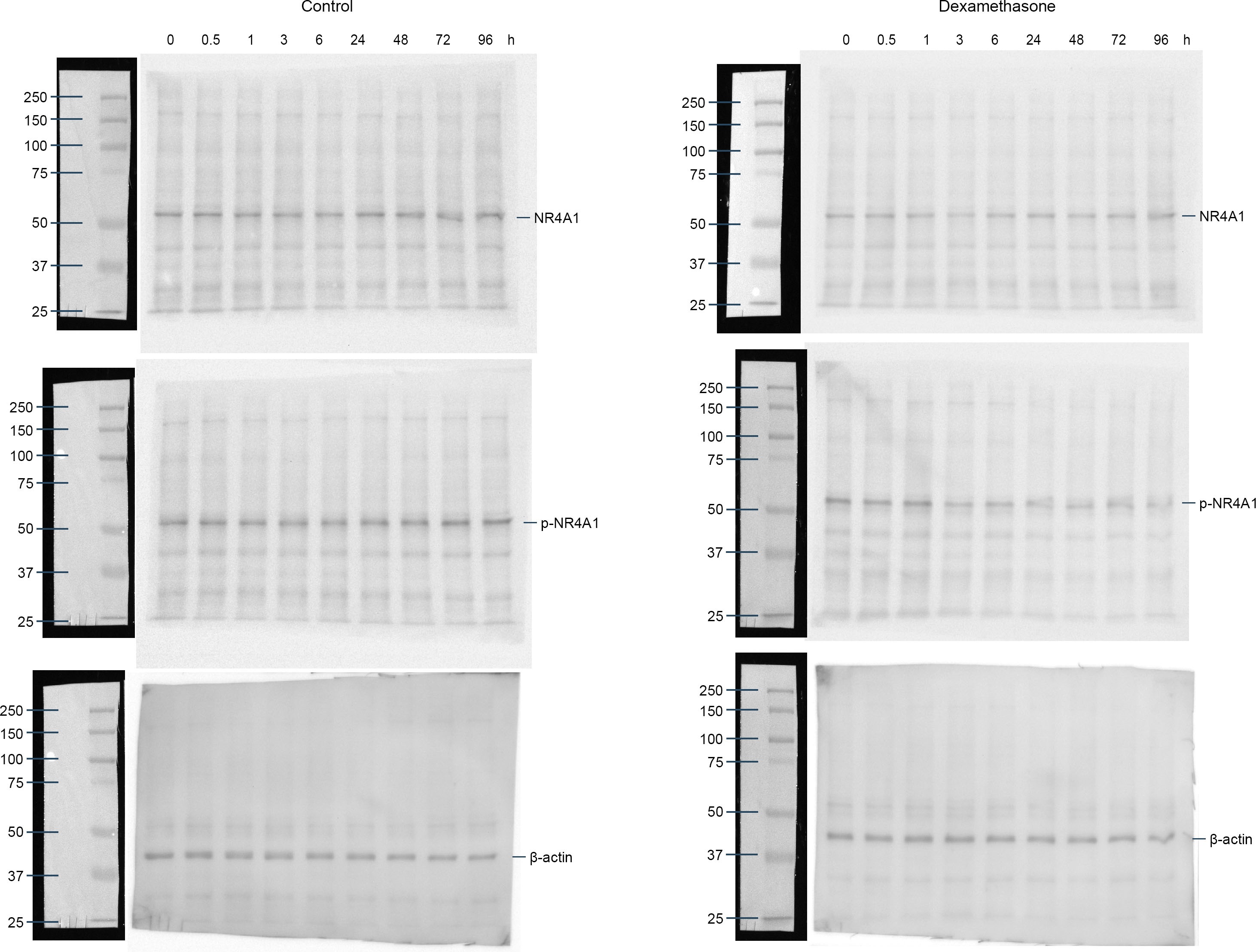

Supplement: Supplementary file 3 — Supplementary Information 3. [file 41598_2020_77445_MOESM3_ESM.jpg]
